# Bioactive Lipids and Circulating Progenitor Cells in Patients with Cardiovascular Disease

**DOI:** 10.5966/sctm.2016-0059

**Published:** 2016-10-14

**Authors:** Salim S. Hayek, Yuri Klyachkin, Ahmed Asfour, Nima Ghasemzadeh, Mosaab Awad, Iraj Hesaroieh, Hina Ahmed, Brandon Gray, Jinhee Kim, Edmund K. Waller, Arshed A. Quyyumi, Ahmed K. Abdel‐Latif

**Affiliations:** ^1^ Division of Cardiology, Emory Clinical Cardiovascular Research Institute, Emory University School of Medicine, Atlanta, Georgia, USA; ^2^ Lexington VA Medical Center and Saha Cardiovascular Research Center, University of Kentucky, Lexington, Kentucky, USA; ^3^ Kasr Alainy Faculty of Medicine, Department of Cardiology, Cairo University, Cairo, Egypt; ^4^ Department of Hematology and Oncology, Winship Cancer Institute, Emory University, Atlanta, Georgia, USA

**Keywords:** Sphingosine‐1 phosphate, Ceramide‐1 phosphate, Circulating progenitor cell, Mobilization, Coronary artery disease

## Abstract

Bone marrow‐derived progenitor cells are mobilized into the peripheral blood after acute myocardial injury and in chronic ischemic heart disease. However, the mechanisms responsible for this mobilization are poorly understood. We examined the relationship between plasma levels of bioactive lipids and number of circulating progenitor cells (CPCs) in patients (*N* = 437) undergoing elective or emergent cardiac catheterization. Plasma levels of sphingosine‐1 phosphate (S1P) and ceramide‐1 phosphate (C1P) were quantified using mass spectrometry. CPCs were assessed using flow cytometry. S1P levels correlated with the numbers of CD34+, CD34+/CD133+, and CD34+/CXCR4+ CPCs even after adjustment for potential confounding factors. However, no significant correlation was observed between C1P levels and CPC count. Plasma levels of S1P correlated with the number of CPCs in patients with coronary artery disease, suggesting an important mechanistic role for S1P in stem cell mobilization. The therapeutic effects of adjunctive S1P therapy to mobilize endogenous stem cells need to be investigated. Stem Cells Translational Medicine *2017;6:731–735*

**Significance Statement**

Bone marrow‐derived progenitor cells are mobilized after acute myocardial injury and in chronic ischemic heart disease. However, the mechanisms responsible for this mobilization are poorly understood. The relationship between plasma levels of the bioactive lipid sphingosine‐1 phosphate (S1P) and circulating progenitor cells was examined in 437 patients undergoing elective or emergent cardiac catheterization. S1P levels correlated with the numbers of CD34+, CD34+/CD133+, and CD34+/CXCR4+ cells, even after adjustment for potential confounding factors. These data suggest an important mechanistic role for S1P in stem cell mobilization that can be explored therapeutically as an adjunctive in future cardiac regenerative studies.

## Introduction

Circulating progenitor cells (CPCs) are mononuclear cells that originate primarily from the bone marrow, differentiate into hematopoietic and endothelial cells, and contribute to vascular repair and cardiomyocyte regeneration [1, 2, 3, 4, 5]. We and others have shown that low CPC counts are predictors of adverse cardiovascular events and are likely biomarkers of regenerative potential [6, 7]. Studies using CPC mobilization as a method to enhance cardiac recovery in heart failure or after myocardial infarction have achieved modest success, probably because of incomplete understanding of the mechanisms underlying mobilization and homing of regenerative cells.

Several chemokines, including vascular endothelial growth factor (VEGF) and stromal cell derived factor‐1 (SDF‐1), play an important role in the recruitment of CPCs from the bone marrow [8, 9]. However, the exclusive role of these chemokines has been debated [10]. After acute myocardial infarction (AMI), matrix metalloproteinases (MMPs) and proteases are upregulated in the myocardium [11], leading to the degradation of several chemokines [12]. More recently, bioactive lipids, notably sphingosine‐1 phosphate (S1P), that are resistant to MMPs have been characterized as chemoattractants that enhance mobilization and homing of stem cells from the bone marrow [13, 14]. Raising plasma S1P levels in a mouse model of AMI increased CPC mobilization and enhanced cardiac recovery [15]. Whether an association between bioactive lipids and CPC counts in patients with coronary artery disease (CAD) exists is unclear and is the subject of this study. We hypothesized that bioactive lipids would mobilize CPCs and thus their levels will be associated with higher counts of CPCs.

## Materials and Methods

### Study Design

We examined the relationship between bioactive lipids and CPCs in subjects (*N* = 437) recruited into the Emory Cardiovascular Biobank, a prospective cohort consisting of patients aged 20 to 90 years who were undergoing elective or emergent cardiac catheterization. Subjects with congenital heart disease, severe valvular heart disease, severe anemia, recent blood transfusion, myocarditis, active inflammatory diseases, and cancer were excluded. Obstructive CAD was defined as >50% luminal narrowing in a major epicardial vessel. The study was approved by the institutional review board at Emory University. All subjects provided written informed consent.

### Quantitation of Bioactive Lipids

Arterial blood was collected in EDTA tubes and plasma was isolated by centrifuging whole blood for 10 minutes at 800*g*. Lipids were extracted from plasma using acidified organic solvents, as previously described [16].

### Measurement of Circulating Progenitor Cells Counts

Peripheral blood mononuclear cells were incubated with fluorochrome‐labeled monoclonal anti‐human mouse antibodies within 4 hours of blood draw. Cell populations enriched for CPCs were quantified using flow cytometry as CD45^dim^ cells coexpressing CD34, CD133, vascular endothelial growth factor receptor 2 (VEGFR2), or CXCR4, and their combination, as previously described [6]. The CD45^dim^ population consists of CD34+ cells with dim expression of CD45, thus excluding hematopoietic CD34+/CD45^med^ and CD34+/CD45^hi^ hematogones or bone marrow‐derived early B‐cell precursors. Flow data were analyzed with FlowJo software (Ashland, OR, http://www.flowjo.com). CPC populations are reported as absolute counts (cells per µl of whole blood), determined using Accucheck Counting Beads (Thermo Fisher Scientific Life Sciences, Waltham, MA, http://www.thermofisher.com).

### Statistical Analysis

Subject characteristics were reported as descriptive statistics with means, medians, standard deviations, and ranges, where appropriate. Correlation analyses were performed using the Pearson’s correlation for normally distributed variables and Spearman’s correlation for non‐normally distributed variables. Cell counts were non‐normally distributed and were transformed (natural log[cell count+1]) before parametric analyses. Multivariable analyses using linear regression of log‐transformed outcomes variables adjusted for age, sex, race (black vs. other), and body mass index, as well variables with a *p* value ≤ .1 on univariable analysis. Analyses were performed using IBM SPSS Statistics version 21 (IBM, Armonk, NY, https://www.ibm.com).

## Results

The majority of enrolled patients were white men; the median age of the patients was 64 years. They had multiple CAD risk factors and 57% had obstructive CAD (Table 1).

**Table 1:** Demographics and clinical characteristics

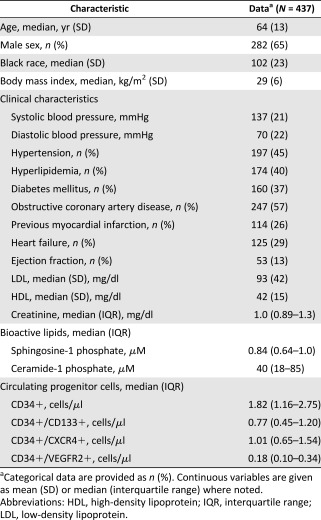

On univariable analyses, plasma S1P and C1P levels were negatively correlated with male sex, history of myocardial infarction, hypertension, hyperlipidemia, and serum creatinine level, whereas they positively correlated with high‐density and low‐density lipoprotein levels. Previous myocardial infarction did not correlate with total C1P levels and was not included in the model. After multivariable adjustments, only the creatinine level and hyperlipidemia remained independently associated with S1P levels (Table 2).

**Table 2:** Independent predictors of bioactive lipids^a^

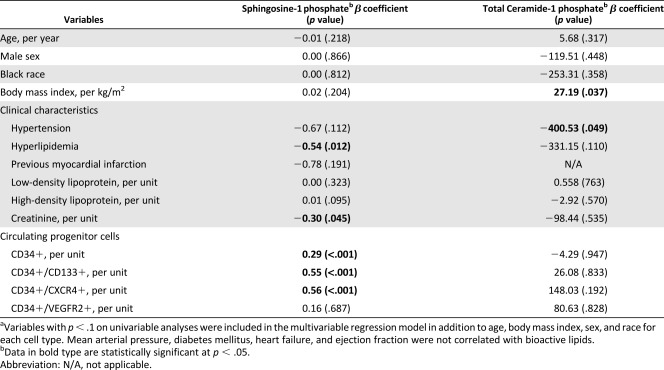

**Figure 1:**
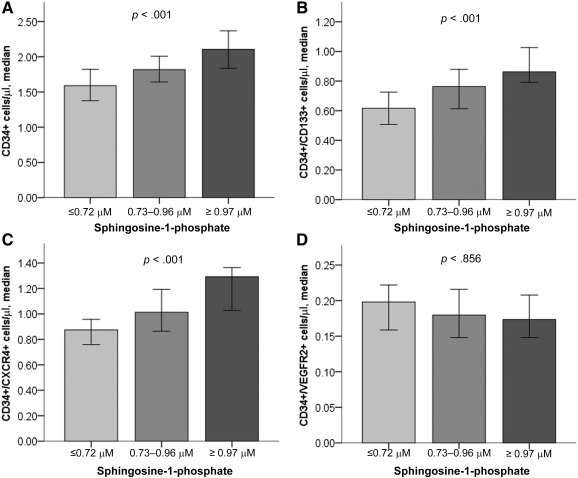
Bar graphs depicting median circulating progenitor cell counts stratified by sphingosine‐1‐phosphate tertiles. Hematopoietic progenitor cell counts for subtypes CD34+ **(A)**, CD34+/CD133+ **(B)**, and CD34+/CXCR4+ **(C)**. **(D):** Cell counts for the endothelial CD34+/VEGFR2+ subtype. Error bars represent 95% confidence interval. Nonparametric Kruskal‐Wallis test *p* values are given.

The number of CPCs (CD45^dim^ cells), in particular CD34+ (*r* = .187; *p* < .001), CD34+/CD133+ (*r* = .217; *p* < .001), and CD34+/CXCR4+ (*r* = .207; *p* < .001) cells correlated positively with S1P levels. However, CD34+/VEGFR2+ cells did not significantly correlate with S1P levels (*r* = −0.30; *p* = .531), and CPC counts did not correlate with C1P levels. On multivariable analysis, S1P levels were significantly associated with the aforementioned CPC populations (Fig. 1; Table 2).

## Discussion

The role of bone marrow‐derived progenitor cells in cardiac repair is still being studied in randomized trials; however, strong evidence suggests that CPCs are mobilized after cardiac injury [17, 18]. In the largest cohort to date examining the relationship between plasma bioactive lipids and circulating stem cells, we demonstrated a strong correlation between plasma S1P levels and the number of CPCs in patients with CAD.

We and others showed that the degree of CPC mobilization correlates with functional cardiac recovery [19] and clinical outcomes [6, 20]. Ischemic myocardial injury activates the immune system, including the complement cascade [21], which plays an important role in the release of bioactive lipids such as S1P from blood [22] and endothelial cells [23], thus increasing S1P’s transendothelial gradient [14]. Of note, number of CD34+/VEGFR2+ cells did not correlate with plasma S1P levels. This could be intrinsic to this cell population, which could be more responsive to VEGF. Interestingly, in an animal model of AMI, temporary elevation of S1P in the plasma resulted in enhanced bone marrow stem cell mobilization and cardiac recovery [15]. In this report, we demonstrated a strong association between plasma S1P levels and mobilized CPCs in a large cohort of patients with CAD, a finding that is in agreement with our report in patients with acute coronary syndrome [14]. Although the present data do not prove a causal relationship between plasma S1P levels and CPC mobilization, combined with findings from our previous mechanistic studies, the results support an important role for S1P in modulating peripheral stem cell trafficking [13].

### Clinical Implications

In conclusion, the bioactive lipid S1P strongly correlated with CPC levels in patients with CAD in this study. Future studies using adjunctive therapy with S1P to enhance bone marrow mobilization of stem cells are needed to investigate its therapeutic benefit.

## Author Contributions

S.S.H.: provision of study material or patients, collection and/or assembly of data, data analysis and interpretation, manuscript writing; Y.K. and A.A.: collection and/or assembly of data, data analysis and interpretation; N.G., M.A., I.H., H.A., B.G., J.K., and E.K.W.: provision of study material or patients, collection and/or assembly of data; A.A.Q.: conception and design, provision of study material or patients, financial support, manuscript writing, final approval of manuscript; A.K.A.‐L.: conception and design, collection and/or assembly of data, financial support, manuscript writing, final approval of manuscript.

## Disclosure of Potential Conflicts of Interest

Y.K. is an employee of and has stock options in Celegene Corp. The other authors indicated no potential conflicts of interest.
